# The Cost of Provisions in Institutions.—XV

**Published:** 1904-08-20

**Authors:** 


### HOSPITAL ADMINISTRATION.

### CONSTRUCTION AND ECONOMICS.

### THE COST OF PROVISIONS IN INSTITUTIONS.—XV.

BY THE AUTHOR OF "HOSPITAL EXPENDITURE—THE COMMISSARIAT."

(*Continued from page* 334.)

#### THE PRICE OF BEEF.

FROM the institution point of view beef is an article of almost equal importance to mutton. In considering its price it falls under the distinct headings of joints for roasting and boiling, and shin for making beef-tea.

We append contract prices for the last few years from the annual reports of the Asylums Committee of the London County Council:—

| Year | Price of Prime Joints of English Beef per stone (14 lb.) | | | | | |
|---|---|---|---|---|---|---|
| | Banstead | | Cane Hill | | Claybury | |
| | March | October | March | October | March | October |
| | s. d. | s. d. | s. d | s. d. | s. d. | s. d. |
| 1895 ... ... | 8 0 | 8 11 | 8 0 | 8 11 | 8 0 | 8 11 |
| 1896 ... ... | 8 2 | 8 0 | 8 2 | 8 0 | 8 2 | 8 0 |
| 1897 ... ... | 8 11 | 8 11 | 8 11 | 8 11 | 8 11 | 8 11 |
| 1898 ... ... | 8 11 | 8 11 | 8 11 | 8 11 | 8 11 | 8 4 |
| 1899 ... ... | 9 4 | 8 6 | 9 4 | 9 6 | 9 4 | 9 6 |
| 1900 ... ... | 9 6 | 9 11 | 9 6 | 9 11 | 9 6 | 9 11 |
| 1901 ... ... | 9 11 | 8 9 | 9 11 | 8 9 | 9 11 | 8 9 |
| 1902 ... ... | 9 11 | 9 0 | 9 11 | 9 0 | 9 11 | 9 0 |

The price quoted for 1902 gives 8½d. for the summer half-year, and 7½d. for the winter half-year, exclusively for prime joints. The prices paid for such joints in London hospitals at the present vary from 10d. to 5½d., according, it may be presumed, to the source of their supply.

The contract prices for legs of beef, English killed, during the last few years are illustrated below:—

| Year | Price of Legs of Beef per stone (14 lb.) | | | | | |
|---|---|---|---|---|---|---|
| | Banstead | | Cane Hill | | Claybury | |
| | March | October | March | October | March | October |
| | s. d. | s. d. | s. d. | s. d. | s. d. | s. d. |
| 1895 ... ... | 4 0 | 5 10 | 4 0 | 5 10 | 4 0 | 5 10 |
| 1896 ... ... | 4 1 | 4 0 | 4 1 | 4 0 | 4 1 | 4 0 |
| 1897 ... ... | 5 11 | 4 10 | 3 8 | 3 8 | 4 8 | 5 3 |
| 1898 ... ... | 4 6 | 4 8 | 4 6 | 4 8 | 4 6 | 4 8 |
| 1899 ... ... | 6 0 | 5 3 | 6 0 | 5 3 | 6 0 | 5 3 |
| 1900 ... ... | 5 8 | 6 6 | 5 8 | 6 6 | 5 8 | 6 6 |
| 1901 ... ... | 6 6 | 5 10 | 6 6 | 5 10 | 6 6 | 5 10 |
| 1902 ... ... | 6 10 | 6 5 | 6 10 | 6 5 | 6 10 | 6 5 |

It will be seen that the price works out at from 5½d. to 6d. per lb.

It is noteworthy that the widest variation exists with regard to the price of shin of beef. Some time ago it was discovered that the following prices per stone of 14 lb. were being paid in London by the different Poor Law Infirmaries for this article:—2s. 11d., 3s. 2½d., 3s. 11d., 4s. 5d., 4s. 10d., 5s. 6½d., 5s. 11¾d., 6s. 0½d., 6s. 5d., 7s. 0½d., 8s. 2d.

These variations, extending between 2½d. and 7d. per lb., are due to the variety of meat which is to be had, and we cannot better illustrate that variety than by quoting the winter prices from the Central Meat Market, Smithfield, for the "Smithfield stone of 8 lb.":—

| Beef. | s. d. s. d. |
|---|---|
| Scotch, short sides ... ... ... | 4 0 to 4 2 |
| " long sides ... ... ... | — — |
| English... ... ... ... ... | 3 2 to 3 6 |
| Cows and bulls (nominal) ... ... | 2 0 to 2 8 |
| American (Deptford killed) ... ... | 3 0 to 3 6 |
| States (Birkenhead killed) ... | 2 11 to 3 4 |
| Ranchers ... ... ... ... | 2 4 to 2 8 |
| American refrigerated, hind-quarters | 3 7 to 3 10 |
| fore-quarters | 2 6 to 2 8 |
| River Plate chilled, hind-quarters ... | 2 8 — |
| " " fore-quarters ... | 1 10 — |
| " frozen, hind-quarters ... | 2 2 — |
| " " fore-quarters ... | 1 7 — |
| New Zealand " hind-quarters ... | 2 4 — |
| " " fore-quarters ... | 1 8 — |
| Australian " fore-quarters ... | 1 5 — |

There are several points worthy of remark in this list of prices, although, of course, being quotations for beef in bulk, they do not indicate the average price of certain joints. It will surprise many people to see that the price of the best American refrigerated beef is higher than that of ordinary English beef. The increase in our outside sources of supply is noticeable, and the varying quality of what is sometimes ignorantly classed all together as "foreign meat," is plainly to be seen by the price it can command.

To ensure a high standard of quality with the lowest standard of price in the supply of beef we must again repeat that knowledge and inspection must go hand-in-hand. The contract should be half-yearly, and should specify the particular joints likely to be needed, with separate prices. It is of the utmost importance that the class of meat required should be specified by name, and that the beef should be under the daily inspection of a trained official able to distinguish unerringly one kind from another.

A low contract for English beef may easily degenerate into the supply of bull or cow-beef, too coarse and tough to be suitable for roasting. A low contract for foreign meat may yield a rich harvest to the contractor, who is permitted to supply Australian beef at a price which may satisfy the managers with a sense of economy, but which is in reality double the market quotation.

The only guide to economy is to test the beef from the various sources of supply—not once, but several times—until a satisfactory standard of quality has been decided on, to ascertain then from independent sources its market value, and to contract for its supply in accordance with facts. The co-operation of a clever housekeeper will be indispensable in carrying out experiments before the contract is decided on, and the inspection we have already indicated is an essential feature in maintaining the standard.

Glancing back once more at Smithfield prices, it will be seen that the price of Australian beef is exactly one-third that of the best English. The ordinary contract price for English ribs of beef would be 7d. per lb.; for the same joint Australian, it should not be more than 3d.; for New Zealand and River Plate, 3d. or 3½d.

Now there is every probability that supplies of frozen meat which are able to command so low a price in the market, although perfectly sound and not deficient in food value, will be found very unsuitable for the hospital table after they have been subjected to the oven process, which passes at the present day for roasting. The writer would be very sorry to be thought to recommend them. But what security does the institution possess that this quality of meat is not supplied in place of the best frozen meat, or even the best English? It may notoriously be often seen in the shops of expensive butchers figuring at a shilling a pound, for there is perhaps no trade in which the easy maxim of *caveat emptor* is more generally acted on by the seller. The only possible way in which the buyer can beware is by learning to know the difference, and until there is one person at least in the institution who does know it, these households will continue to suffer from the standing grievance of tough meat.

Investigations carried out some years ago with regard to the food value of beef-tea, made with the cheapest foreign and best English meat respectively, proved to the satisfaction of all concerned in them that the foreign meat presented no disadvantages from this point of view. We have, therefore, no hesitation in recommending the cheap Australian or River Plate shin of beef for the preparation of beef-tea. It is noteworthy that in some hospitals as low a price as 2¾d. per lb. is paid for shin of beef, while in others it is contracted for at from 4d. to 6d. per lb.

Although dealing with beef in bulk cannot be attempted in a general way, it would, we may suggest, be to the advantage of large institutions in which several hundred pints of beef tea are required every week, to contract for a fore-quarter of beef weekly. Roughly speaking, the fore-quarter from which the shin is cut weighs about 2 cwt. Of this amount about 1 cwt. would cut up into joints, ribs, and brisket, very suitable for stewing and braising, in which latter form, especially, it would prove very palatable and easy of digestion. The remaining hundredweight, including about 15 lb. of bone, would be available for beef-tea. The cost would work out somewhat as follows:—

Two hundredweight of beef at, say, 2s. per stone, allowing for expenses of delivery, will cost £2 16s. Of this one hundredweight will cut into joints worth, at the lowest computation, 4d. per lb., or £1 17s. 4d. in all. The remaining 9s. 4d. brings the cost of the hundredweight of shin to exactly 2d. a lb., including the bone which, however, if used as it should be in the preparation of the beef-tea, would not be wasted. The difficulties in the way of cutting up the meat are often much exaggerated. A dexterous man would manage it in the course of an hour, and would need only an ordinary meat block, saw and hatchet. The very large extent to which beef-tea is used in hospitals renders it a matter of obvious importance to secure a cheap source of supply for its preparation.

We have not done more than touch the fringe of this big question—the meat supply to institutions.

It can only be dealt with effectually by the establishment of a co-operative meat store under the control of the hospitals, and managed in their interests, and of the starting of such an establishment there are no signs at present. Meantime, the energy of individuals may accomplish much, both in levelling prices and in raising the quality of the supplies.

